# Right Thoracoscopic Excision of an Esophageal Bronchogenic Cyst

**DOI:** 10.7759/cureus.66119

**Published:** 2024-08-04

**Authors:** Zoi Nitsa, Prodromos Kanavidis, Pagona Kastanaki, Stelios Faltsetas, Alexandros Charalabopoulos

**Affiliations:** 1 First Department of Surgery, Laiko General Hospital of Athens, Athens, GRC; 2 First Department of Surgery, Saint George General Hospital of Chania, Chania, GRC; 3 Department of Upper Gastrointestinal Surgery, University of Athens, Athens, GRC; 4 First Department of Surgery, National and Kapodistrian University of Athens, Athens, GRC

**Keywords:** video-assisted thoracoscopic surgery (vats), esophageal stricture, minimally invasive surgical procedures, esophageal bronchogenic cyst, right thoracoscopy

## Abstract

Bronchogenic cysts, first described in 1859, are rare congenital cystic malformations of the respiratory tract, with an incidence of one per 42,000-68,000 hospital admissions in one hospital series. They comprise 10-15% of mediastinal tumors and between 50% and 60% of mediastinal cystic lesions. Its clinical diagnosis is often challenging due to the absence of distinct imaging features. This case report focuses on the case of a 51-year-old female who initially received a misdiagnosis of esophageal leiomyoma. Subsequently, during exploration in the operating theater, right thoracoscopy revealed the presence of an esophageal bronchogenic cyst.

## Introduction

Bronchogenic cysts are lesions of congenital origin derived from the primitive foregut [1] and are the most common primary cysts of the mediastinum. Most frequently unilocular, they contain clear fluid or, less commonly, hemorrhagic secretions or air [2]. They are lined by columnar ciliated epithelium, and their walls often contain cartilage and bronchial mucous glands [3,4]. It is unusual for them to have a patent connection with the airway, but when present, such a communication may promote infection of the cyst by allowing bacterial entry. Most bronchogenic cysts originate in the mediastinum, while 15-20% occur in the lung parenchyma [3-5]. According to the literature, most intrapulmonary cysts occur in the lower lobes [3,5].

## Case presentation

**Figure 1:**
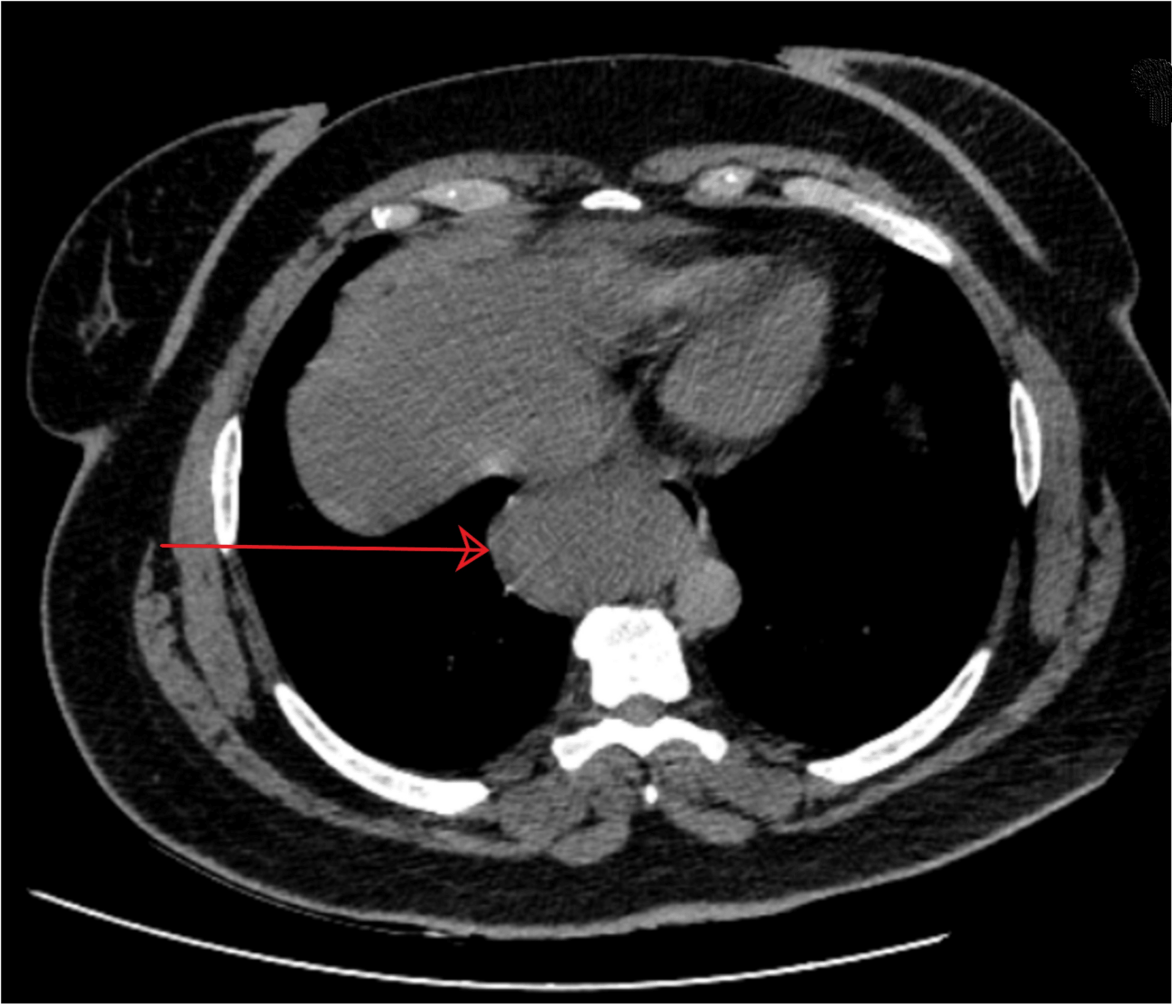
Submucosal tumor of the lower esophagus extending to the right part of the mediastinum Axial plane of thoracic computed tomography (non-contrast)

**Figure 2:**
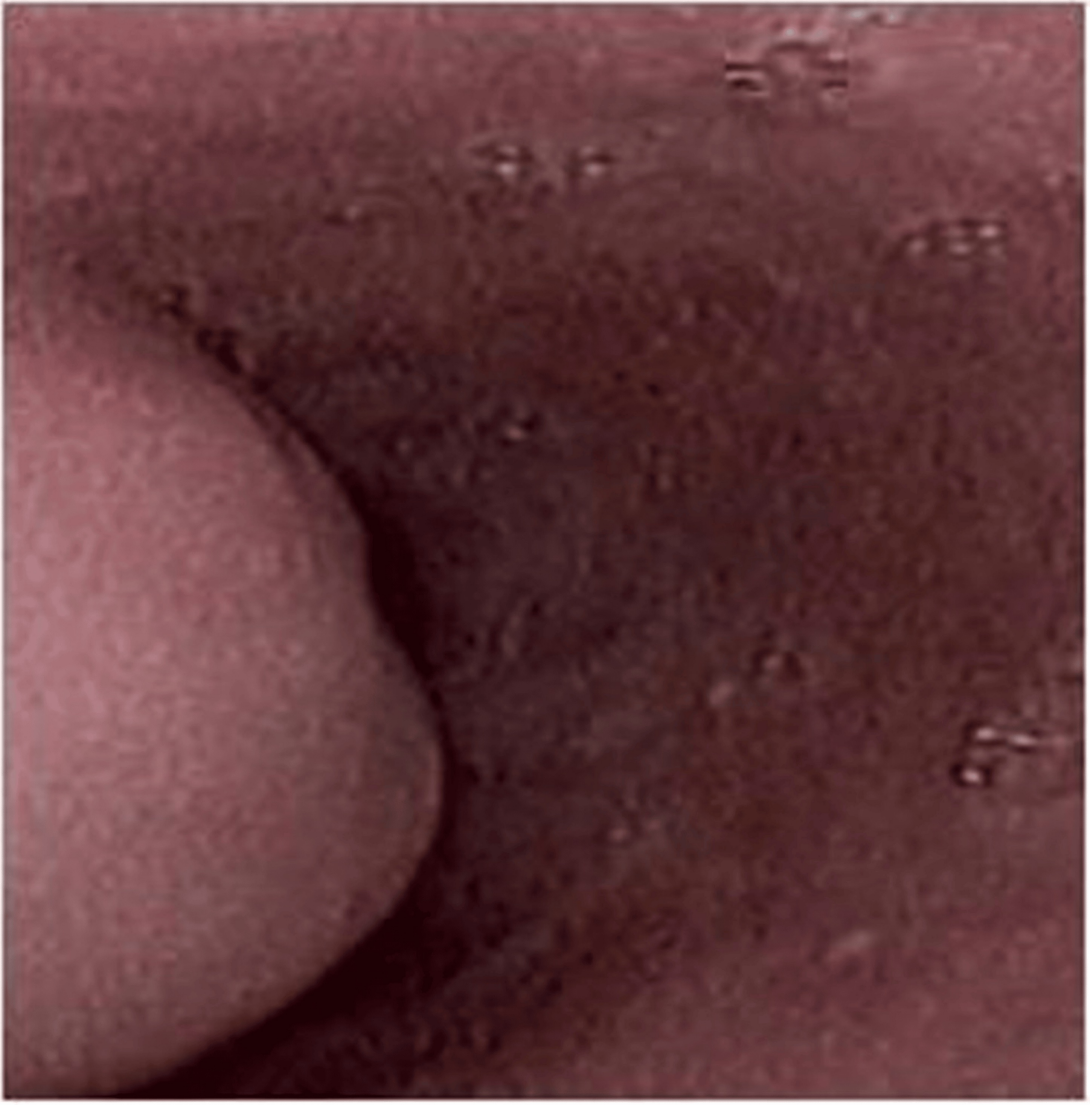
Upper gastrointestinal endoscopy revealing a submucosal protruding lesion with an intact mucosal surface and a normal color

A 51-year-old female with mild comorbidities (controlled hypertension; American Society of Anesthesiologists (ASA) II) presented with a persistent six-month history of cough and dysphagia. Physical and laboratory examinations showed unremarkable results. A thoracic computed tomography (CT) scan unveiled a tumor in the lower right part of the esophagus extending to the mediastinum (Figure 1). Upper gastrointestinal endoscopy (Figure 2) and endoscopic ultrasound were performed and revealed a submucosal tumor indicative of an esophageal leiomyoma. The patient was led to the operating theater and a right thoracoscopy was performed.

**Figure 3:**
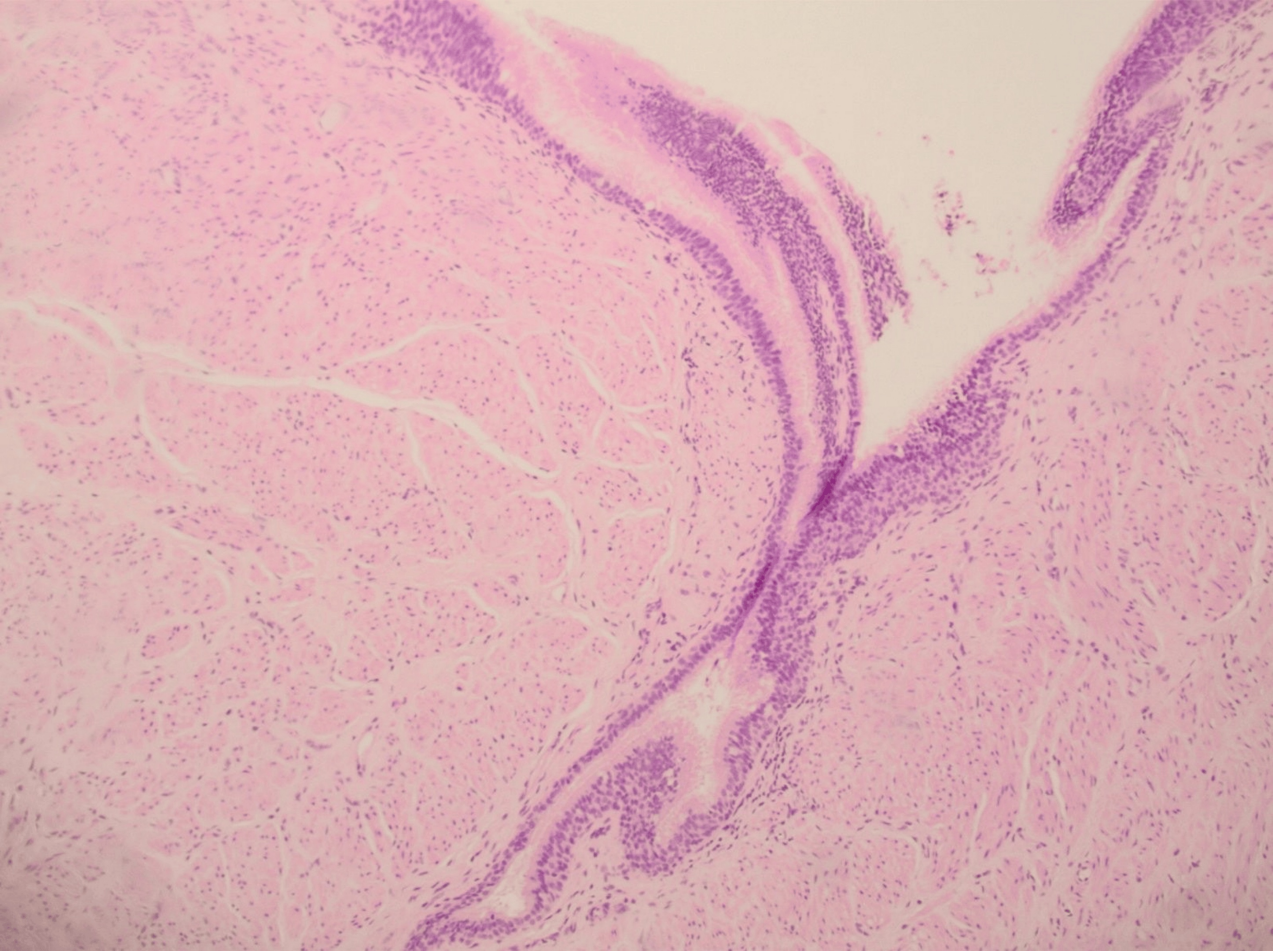
High-magnification micrograph of the esophageal bronchogenic cyst

Intraoperatively, it was discovered that the tumor was filled with fluid, a finding inconsistent with the initial diagnosis of leiomyoma. The fluid was aspirated and sent for cytologic evaluation. The remaining cyst, which did not communicate with the esophageal lumen, was excised and sent for final histopathology, revealing an esophageal bronchogenic cyst (Figure 3). The operation lasted 90 minutes, with minimal blood loss. The patient had an uncomplicated postoperative course, was started on clear fluids on postoperative day 1, and was discharged on postoperative day 4. As of 12 months post-surgery, the patient remains in good health with no recurrent symptoms.

## Discussion

A bronchogenic cyst is very rare with an incidence of approximately 0.5-1 per 50,000 admissions. It is a malformation of the respiratory tract, which embryologically is derived from the foregut [2]. It develops within the mediastinum in the lungs and foregut even in an ectopic site [6]. Depending on the location of the cyst, there are five types: paratracheal, carinal, paraesophageal, hilar, and mixed [7]. In the majority of cases, bronchogenic cysts are located intrapulmonary. Our case involves an intramural bronchogenic cyst, which is a very rare occurrence, as these cysts typically manifest as intraparenchymal [8]. Extremely rare positions where it can develop are the subcutaneous tissue of the chest wall, scapula, and paravertebral, cervical, retroperitoneal, pericardial, omental, and perianal regions [9]. Bronchogenic cysts usually contain clear fluid, while in rare cases they contain air or hemorrhagic secretions and are typically unilocular [10]. Regardless of the location, bronchogenic cysts need to be completely excised (R0) not only for symptomatic patients but for those asymptomatic too, because there are a high incidence of recurrence and a potential malignant transformation such as bronchioalveolar carcinoma, adenocarcinoma, squamous cell carcinoma, and melanoma [11-13]. The recurrence time of R1-R2 resection of a bronchogenic cyst is estimated to exceed 20 years. Complications associated with cysts involve rupture into the airway, bleeding, and infection [14]. The resection of those cysts can be performed surgically or endoscopically. An endoscopic resection would be a less complicated technique than surgical resection in specific cases of intramural and small cysts. It is crucial for the diagnosis of the bronchogenic cyst to be done preoperatively in order to form the most effective treatment plan [15]. Bronchogenic cysts can in fact mimic hydatid cysts. However, the CT density reading may be higher, comparable to that of soft tissue, which can create other problems in diagnosis. Also, the cysts may have an air/fluid level that is visible upon radiography. Occasionally, pneumonitis, pneumothorax, or empyema is apparent, as in our series [14]. Endoscopic ultrasound-guided fine-needle aspiration (EUS-FNA) is a technique which allows the study of cells obtained through aspiration in different locations near the gastrointestinal tract. EUS-FNA is used to acquire tissue from mucosal/submucosal tumors, as well as peri-intestinal structures including the lymph nodes, pancreas, adrenal gland, gallbladder, bile duct, liver, kidney, lung, etc. Except for CT scan or magnetic resonance imaging (MRI), EUS-FNA should be also done but without being advocated [15].

## Conclusions

With regard to the management of esophageal bronchogenic cysts, complete surgical removal by thoracotomy or video-assisted thoracoscopy is recommended, even when they are asymptomatic, because of subsequent complications of infection, rupture, intracystic hemorrhage, and carcinomatous change. As in our case, the intramural cyst may be associated with greater difficulty in resection due to the lesion length. Endoscopic submucosal tunnel dissection (ESTD) is a newly effective and safe procedure to treat submucosal tumors originating from the muscularis propria and has been successful in extracting an intraesophageal bronchogenic cyst. This treatment would be a less complicated and less risky choice, but long-term follow-up visits and complications are required to be evaluated further. EUS seems to be a valuable option for diagnosis and surveillance.
